# Mitochondrial Genes of Dinoflagellates Are Transcribed by a Nuclear-Encoded Single-Subunit RNA Polymerase

**DOI:** 10.1371/journal.pone.0065387

**Published:** 2013-06-19

**Authors:** Chang Ying Teng, Yunkun Dang, Jillian C. Danne, Ross F. Waller, Beverley R. Green

**Affiliations:** 1 Botany Department, University of British Columbia, Vancouver, B.C., Canada; 2 Life Sciences Department, Ludong University, Yantai, Shandong, China; 3 School of Botany, University of Melbourne, Melbourne, Victoria, Australia; King Abdullah University of Science and Technology, Saudi Arabia

## Abstract

Dinoflagellates are a large group of algae that contribute significantly to marine productivity and are essential photosynthetic symbionts of corals. Although these algae have fully-functioning mitochondria and chloroplasts, both their organelle genomes have been highly reduced and the genes fragmented and rearranged, with many aberrant transcripts. However, nothing is known about their RNA polymerases. We cloned and sequenced the gene for the nuclear-encoded mitochondrial polymerase (RpoTm) of the dinoflagellate *Heterocapsa triquetra* and showed that the protein presequence targeted a GFP construct into yeast mitochondria. The gene belongs to a small gene family, which includes a variety of 3′-truncated copies that may have originated by retroposition. The catalytic C-terminal domain of the protein shares nine conserved sequence blocks with other single-subunit polymerases and is predicted to have the same fold as the human enzyme. However, the N-terminal (promoter binding/transcription initiation) domain is not well-conserved. In conjunction with the degenerate nature of the mitochondrial genome, this suggests a requirement for novel accessory factors to ensure the accurate production of functional mRNAs.

## Introduction

Dinoflagellates are a large and diverse group of algae, which are major contributors to marine primary productivity and include the essential endosymbionts of corals [1], [2]. They include both photosynthetic and non-photosynthetic species, some of which have parasitic life-styles. Their nuclear genomes are extremely large, with permanently condensed chromosomes containing unusually low levels of histones and other organizing proteins [3]. In contrast, the genomes of both the mitochondrion and the plastid are highly reduced and uniquely organized. In both photosynthetic and non-photosynthetic dinoflagellates, the mitochondrial genomes carry genes for only three essential components of the electron transport chain (*cob*, *cox1* and *cox3*) plus fragments of rRNA genes [4]–[9]. The protein-coding genes are organized on short linear molecules with a variety of flanking sequences. Gene fragments outnumber complete sequences, and are often linked to other partial sequences and gene fusions. The chloroplast genomes of photosynthetic dinoflagellates are also reduced, encoding no more than 20 core genes of the photosynthetic electron transport chain plus two rRNAs and several tRNA genes [10]–[12]. The genes are organized on 2–5 kb minicircles carrying from one to five genes, and in several species there is evidence for minicircles consisting of fragments of several genes [12], [13]. Both of the dinoflagellate organelle genomes have clearly been subjected to massive gene rearrangement in the course of reduction.

Nothing is known about the RNA polymerases that transcribe the organellar genomes of dinoflagellates. In almost all eukaryotes, mitochondrial transcription involves a nuclear-encoded single-subunit RNA polymerase related to the bacteriophage T7 RNA polymerase [14]. The one exception is found in the largest mitochondrial genome, that of the protist *Reclinomonas*, which encodes a multisubunit RNA polymerase of the proteobacterial type [15]. In all other eukaryote lineages, this polymerase was replaced by the bacteriophage type early in eukaryote evolution [16]. In contrast, chloroplasts of photosynthetic eukaryotes have retained a chloroplast-encoded multisubunit RNA polymerase inherited from their cyanobacterial ancestor [17]–[19]. In addition, chloroplasts of higher plants and moss employ a second RNA polymerase, a nuclear-encoded single subunit polymerase (RpoTp) related to that of the mitochondrial polymerase and probably derived via an ancient duplication of the mitochondrial polymerase gene (*RpoTm*). However, all the algal genomes sequenced to date have just one T7-type polymerase gene which encodes the mitochondrial RpoTm, and their chloroplasts use only the cyanobacterial-type polymerase.

In this study, we looked for organellar RNA polymerases in the photosynthetic dinoflagellate *Heterocapsa triquetra and* found a nuclear gene encoding a single subunit RNA polymerase of the RpoTm type. Its N-terminal targeting sequence was able to direct GFP into yeast mitochondria [20], supporting its identification as a mitochondrial RNA polymerase. The protein sequence shares the nine conserved sequence blocks found in the C-terminal (catalytic) domain of other polymerases of this type [14] but shows little relatedness in the N-terminal domain, which is involved primarily in promoter recognition and initiation of transcription [21], [22].

## Materials and Methods

### Algal Culture

An axenic culture of *Heterocapsa triquetra* (CCMP 449) was obtained from the Provasoli–Guillard Culture Center for Marine Phytoplankton (Boothbay Harbor, ME) and grown in seawater supplemented with f/2–Si nutrients [23] at 18° and 50 µmol photons m^−2^ s^−1^ light on a 12-h light/12-h dark cycle. Cells were subcultured to maintain them in exponential phase (0.66 divisions per day) and harvested before they reached 0.8×10^4^ cells/mL.

### Sequence of the Conserved C-terminal Domain of rpoT

*H. triquetra* cells were collected by centrifugation and broken with a Mini-Beadbeater (Biospec) at 4800 rpm for 1 minute with 0.1 mm-diameter beads. Total RNA was extracted with RNAqueous –4 PCR Kit (Ambion), then treated with Dnase I (Invitrogen) for 30 minutes. For cDNA synthesis, 2µg of total RNA was transcribed with Super Script III –RNase H Reverse transcriptase (Invitrogen) using random hexamers as primers (Invitrogen).

The degenerate primers for *rpoT* cloning (Table S1) were adapted from Cermakian et al. [14]. The first round PCR was carried out with rpoTdf1 and rpoTdr for 35 cycles: 94°C for 30 s, 53°C for 1 min, 56°C for 20 s, 60°C for 5 s, followed by 72°C for 2 min. A 0.7 kb band was recovered from the gel, diluted 100 times and used as template for a second round of PCR with rpoTdf2 and rpoTdr primers. Product was gel purified and sequenced directly. Based on the sequence of the product, 5 gene specific inverse primers were designed (rpoTinvf1–3, rpoTinvr1–2) and used for nested PCR using cDNA derived from mRNA circularized with T4 RNA ligase [24].The 5′ end sequence was extended by two sequential 5′-RACE reactions [25], first using gene specific primers rpoTr 1–3, which gave a 0.6 kb product and then primers rpoTr4–6 which gave a 0.7 kb product. PCR products were either directly sequenced or cloned into plasmids with TOPO TA cloning Kit (Invitrogen) for sequencing. The combined sequences totalled 2.1 kb and included the complete 3′ end of the rpoT ORF.

### 5′- RACE using Spliced Leader Sequence

For RNA isolation, about 7×10^7^ cells were collected by centrifugation then frozen and ground in a mortar and pestle with liquid N_2_. The cell powder was suspended in 10 mL Trizol (Ambion) and incubated at room temperature for 5 min before being shaken vigorously with CHCl_3_ for 30 sec, then spun at 12,000×g for 15 min. The supernatant was removed and combined with an equal volume of 70% ethanol. Subsequent steps employed the RNAqueous –4 PCR Kit (Ambion) according to the manufacturer’s instructions. RNA was eluted in 50 uL RNAase-free water and treated with Dnase I (Invitrogen) for 30 minutes.

Reverse transcription and PCR were performed using Invitrogen Thermoscript RT-PCR Kit. Reverse transcription was done with 2 ug of RNA (DNA free) at 55°, with Thermoscript RT as reverse transcriptase, using random hexamers as primers. Two rounds of PCR were done using Platinum Taq polymerase and kit provided buffer plus 2 uL of DMSO per 50 uL of reaction volume. First round PCR was done with 2 uL of first strand cDNA primer r81 paired with Uni5′Ht, and the second round was done by using 100 times diluted first round product with primer r82 and Uni5′Ht. The gene-specific reverse primers for PCR (Table S2) were designed based on the previously obtained incomplete 5′-end sequence. The forward primer Uni5′Ht was synthesized according to the conserved spliced leader sequence of dinoflagellates [26]. The program for the first round PCR was 95° for 80 sec, 62° for 1 min, 70° for 2 min; followed by 10 cycles of touchdown PCR, starting with 95° for 20 s, 70° for 30 s, 70° for 2 min and decreasing the annealing temperature by 1° every cycle. This was followed by 25 cycles of 95° for 20 s, 60° for 30 s, 70° for 2 min; and a final elongation at 72°C for 10 min. For the second round of PCR, the program was similar to the first round, except for the first 3 steps.The resulting sequence including the spliced leader was combined with the previous sequence, giving a final length of 3563 nt (Genbank accession number: GU390406).

### 3′-RACE for Alternative Ends

First strand cDNA was synthesized from 2 ug of total RNA (DNA free) using First Choice RLM-RACE Kit and the supplied T-tailed 3′ RACE Adaptor. Five pairs of outer and inner forward primers, p2f1/3′endf1, f1200/p1f1, p1f1/f2050, f2050/f2300 and f2300/f2550 (Table S2), based on the previously obtained 3′ sequence, were used to do five separate two-round PCRs. All the amplifications were done with the Invitrogen Plantinum Taq polymerase kit with the addition of 2 uL DMSO/50 uL reaction. PCR programs for all the amplifications employed 10–14 cycles of touchdown PCR with primer Tm±2 as start annealing temperature, decreasing by 0.5° per cycle, followed by 28–30 cycles with annealing temperatures 3–7° lower than Tm. The products were cloned into pGEM-T plasmid vectors and randomly selected colonies were sequenced.

### Genomic DNA Sequences

Extraction of total genomic DNA followed the methods described in Zhang et al. [12], except that DNA was not purified with the CsCl-Hoechst dye density gradient fractionation. PCR was done with 100 ng genomic DNA per 50 uL reaction, and various combinations of primers (Table S1). For two of the genomic PCRs, touchdown PCR was employed, with annealing temperature decreased from 69 to 62 for primer pairs p2f1/p2r1, or 70° to 63° for f2750/r3100. For the PCR primed by f2550/r2750 and the genomic PCR for transcript variants, regular PCR was done with annealing temperature at 58° for f2050/3FRC13-r1 and f2750/3FRC11-r1, 62° for 3RACE10-f1/3RACE10-r1, and 66° for f2300/3FRC33-r1. All the PCR products were gel-isolated and then cloned into the plasmid pGEM-T(Promega) for sequencing.

### Immunoblotting

Protein samples were separated on SDS-PAGE gels, transferred electrophoretically to nitrocellulose membranes (Amersham) and blocked with 5% milk in Tris-buffered saline containing 0.05% Tween 20 for at least 1 hr. Blots were first incubated with 1∶1000 dilution of anti-Zm-RpoTp [27] (gift of Dr. D. Stern) and then with a 1∶10,000 dilution of commercial peroxidase-linked secondary antibodies. Signals were detected with the chemiluminescence system (Amersham).

### Yeast Transformation

The *RpoT* gene sequence corresponding to the first 30 residues was amplified and appended upstream of the gene for GFP by spliced- overlap extension PCR [28]. This fusion gene was cloned into the pYES2.1/V5-His-TOPO (Invitrogen) vector according to the manufacturer’s instructions and verified by DNA sequencing. Haploid *S. cerevisiae* strain MH272 was transformed and plated onto uracil- deficient selective medium [2% (wt/vol) agar, 2% (wt/vol) glucose, and 0.67% (wt/vol) yeast nitrogen base supplemented with relevant amino acids]. Positive colonies were grown for 2 days on uracil-deficient SD plates with 2% (wt/vol) galactose for fusion gene induction. Live cells were imaged for GFP localization using a Leica TCS SP2 laser scanning confocal microscope (Wetzlar, Germany). Co-localisation in chemically fixed cells was performed by immunofluorescence assay [29] using an antibody raised to the β subunit of ATP synthase (AS05 085, Agrisera) and goat anti-rabbit Alexa-Fluor 594.

### Phylogenetic and Structural Analysis

Sequences related to the dinoflagellate mitochondrial RpoT were obtained by searching public databases. They included two unannotated partial *RpoT* transcripts from the dinoflagellate *Lingulodinium polyedrum* [30]. Amino acid sequences were aligned with MAFFT [31] using the E-ins option and the JTT matrix, and the alignments were refined using BioEdit [32] after assessment with Gblocks [33]. Maximum likelihood trees were generated using the PhyML web-server (http://www.atgc-montpellier.fr/phyml/) with the LG option, and visualized with TreeView (ver 1.6.6). Accession numbers are given in Table S3.To model the C-terminal domain, we used the Phyre2 web-server [34] http://www.sbg.bio.ic.ac.uk/phyre2.

## Results

### A Phage T7 type RNA Polymerase

**Figure 1:**
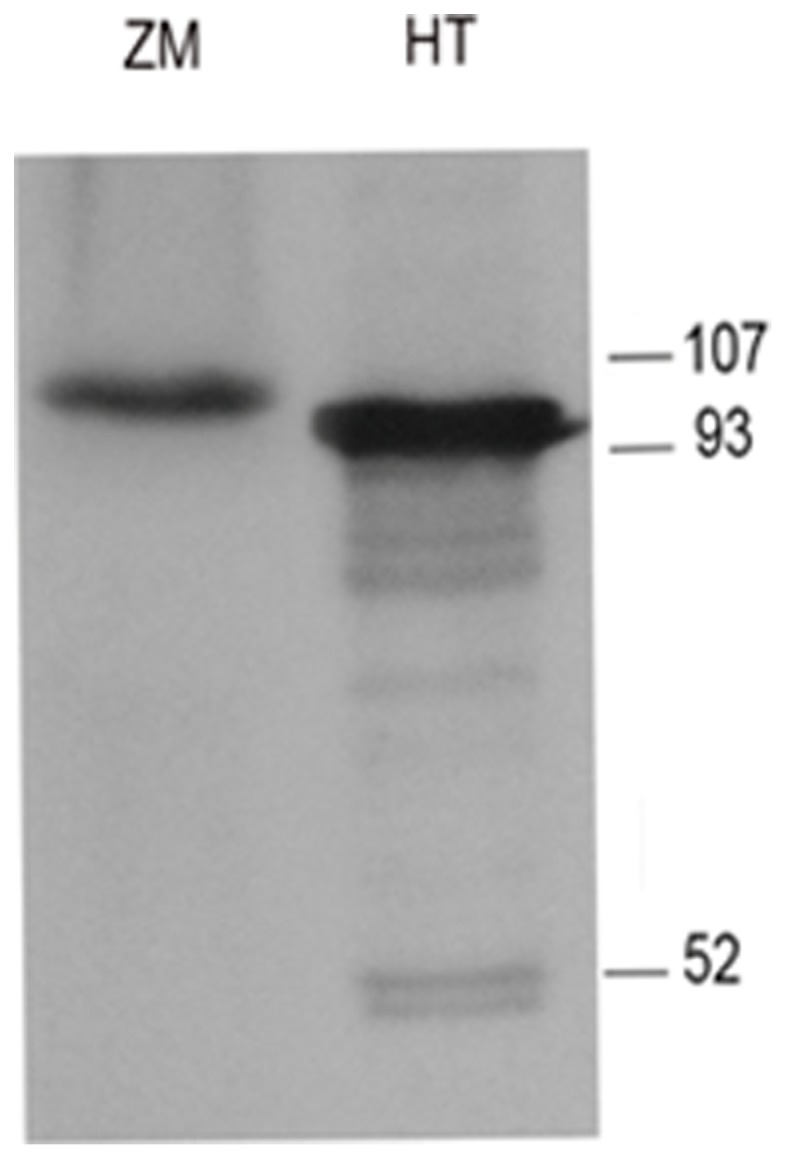
Immunostaining of *H.* *triquetra* RpoT-type RNA polymerase. Immunoblot of *H. triquetra* and *Zea mays* proteins with antisera specific for maize RpoTp. Proteins (20 ug of total soluble protein) were separated on 8% SDS-PAGE, blots were incubated with 1∶1000 dilution of anti-Zm-RpoTp and immunostaining was visualized with the ECL system (Amersham). HT, *H. triquetr*a; ZM, *Z. mays*.

In higher plants and bryophytes, the mitochondrial bacteriophage-like RNA polymerase gene (*rpoTm*) has been duplicated and its product retargeted to the chloroplast, where it is responsible for transcription of some housekeeping genes [18]. Some plants have additional members of the family that are dual-targeted to both organelles [35], [36]. Since no plastid RNA polymerase genes have been reported from dinoflagellates, this raised the possibility that nuclear-encoded *RpoT* genes could be responsible for transcription in both organelles of these algae. The C-terminal half of RpoT is conserved across a wide spectrum of species [14], [37], so we used an antibody raised against a peptide consisting of 100 amino acids at the C-terminus of maize rpoTp [27] to probe a blot of *H. triquetra* and *Z. mays* total protein (Fig. 1). The *H. triquetra* lane showed a strong band of about 98 kDa, just slightly less than the *Z. mays* band, indicating that there is at least one RpoT-type RNA polymerase in the dinoflagellate.

By using degenerate PCR with primers adapted from Cermakian et al. [14] based on the conserved C-terminal half of known *rpoT* genes, and a cDNA template, a 0.7 kb product with a sequence clearly related to that of the other *rpoT* genes was obtained from *H. triquetra*. The sequence was extended in both directions with cDNA based inverse PCR to give a 2.1 kb sequence, which included the complete 3′-end of the *rpoT* open reading frame. The 5′ end of the gene was further extended by 5′ RACE up to a total sequence length of 3052 bp, but could not be extended further.

The sequence encoded an open reading frame of 954 amino acids (106 kDa) corresponding approximately to the size of the band on the immunoblot. However, analysis of the first 100 amino acids with SignalP [38] and other prediction programs did not show any leader sequence that would target it to either mitochondria or chloroplasts.

### The 5′-end of rpoT Encodes a Mitochondrial Targeting Sequence

Dinoflagellate nuclear transcripts start with a common spliced-leader sequence that is added onto the 5′ end of the mRNA in a post-translational step [26]. Using an improved protocol for RNA isolation, random hexamer primed first-strand cDNA synthesis and a different program (Methods), we obtained a band of about 650 bp in two rounds of nested PCR using spliced-leader forward primer Uni5′Ht and gene-specific reverse primers r81 and r82 (Table S2). The product was first gel-isolated and then cloned into a T-A plasmid vector and 24 independent colonies were randomly selected for sequencing, all of which had the same sequence. The 3′ end overlapped with the previously obtained sequence by 150 bp and the 5′ end included the 22 bp spliced leader. The assembled sequence was confirmed by PCR sequencing across all the joins using first strand cDNA as template.

**Figure 2:**
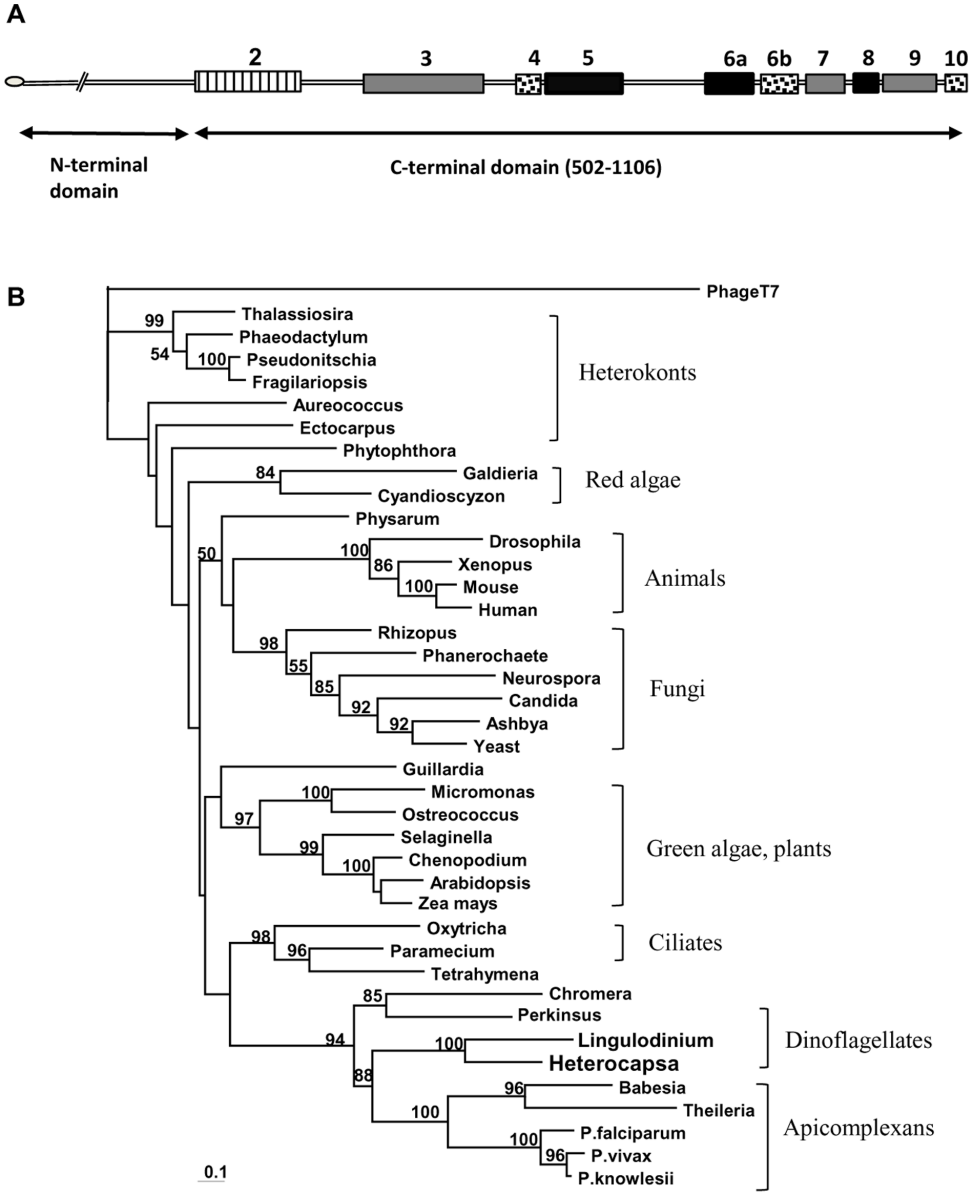
*H.* *triquetra* mitochondrial RNA polymerase. A. Schematic of the RpoT structure derived from multiple sequence alignments. Boxes indicate the conserved sequence blocks in the C-terminal domain (residues 502–1106), numbered following [37]. No region comparable to Block 1 of [40] could be identified in the N-terminal domain. Black blocks, 35–37% amino acid identity; gray blocks, 20–27% identity, dotted blocks, less than 20% identity but more than 25% similarity. Although block 10 has only three conserved residues, one of them is the penultimate Phe822 which is essential for nucleotide binding [21]. Block 2 (striped) consists of several smaller blocks of sequence relatedness. Lines (not to scale) represent regions with variable lengths and little or no relatedness among the RpoTs of different organisms. Grey oval, mitochondrial targeting sequence. B. Maximum likelihood phylogenetic tree of mitochondrial RNA polymerases plus that of bacteriophage T7, based on alignment of Blocks 3–10. Block 2 was not included because the T7 sequence was too divergent to make a reliable alignment. Full names and relevant accession numbers are given in Table S3. The *Lingulodinium* sequence was derived from two separate transcripts deposited in the Genbank TSA archive.

The final *RpoT* gene sequence had a length of 3563 bp including the spliced leader, and was 63.7% G+C, close to the average for *H. triquetra* ESTs [39]. It gave an open reading frame of 1106 amino acids in length. Alignment of the deduced protein sequence with homologs downloaded from Genbank and JGI showed that it had all nine of the conserved sequence blocks found in the catalytic C-terminal domain of the protein structure [14], [37], [40] but was quite divergent between these blocks (Fig. 2A). There was very little sequence relatedness in the N-terminal domain of the protein, which is largely involved in promoter binding and initiation of transcription [21], [22].

To detect a possible mitochondrial targeting motif, the deduced N-terminal protein sequence was submitted to the online programs iPSORT (http://ipsort.hgc.jp/) and TargetP (http://www.cbs.dtu.dk/services/TargetP/), which both gave a positive prediction for mitochondrial targeting. The first 30 residues of the targeting sequence were enriched in hydroxylated residues, had no acidic resides and had a net positive charge of +5. The first 40 residues (the cleavage site predicted by TargetP) had a net charge of +7. These properties are consistent with other dinoflagellate mitochondrial sorting signals [20]. The program SignalP (http://www.cbs.dtu.dk/services/SignalP/) did not detect an ER signal peptide, which would be required for a protein to be translocated into the secondary plastid of a dinoflagellate [41].

**Figure 3:**
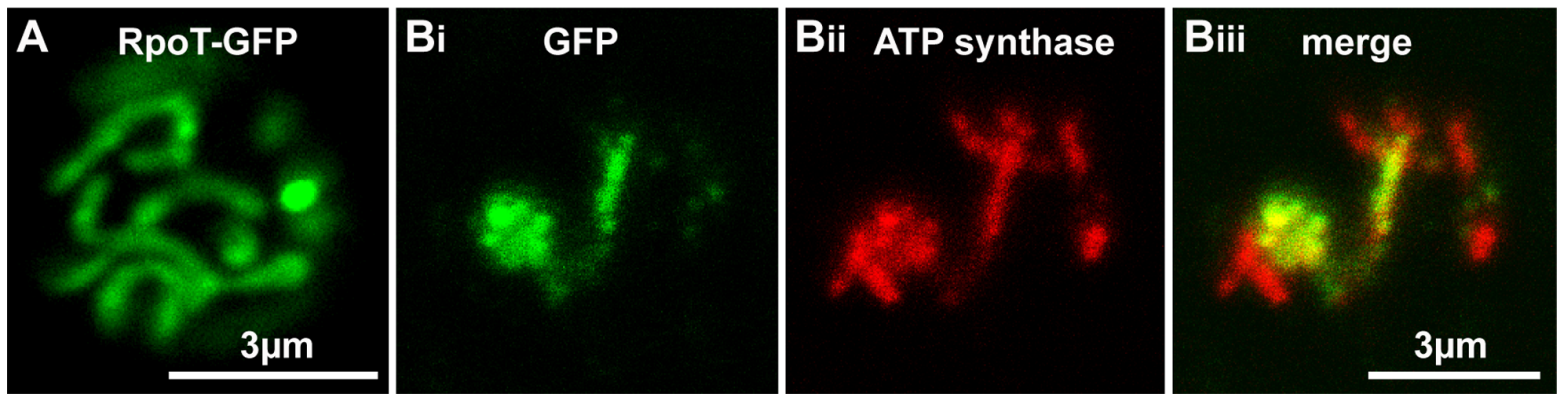
RpoT-GFP localises to the *S.* *cerevisiae* mitochondrion. *A*. Single optical section of a live cell transformed with a construct consisting of the first 30 amino acids of *H. triquetra* RpoT followed by GFP, showing its location in the mitochondria. *B*. Chemically fixed GFP-expressing cellls showing co-localization of (i) RpoT-GFP (green) (ii) mitochondrial ATP synthase immunofluorescence (red) and (iii) the overlay of these two signals.

It has previously been shown that the presequences of nuclear-encoded mitochondria-targeted dinoflagellate proteins can correctly target reporter constructs into yeast mitochondria [20]. When the first 30 amino acids of *H. triquetra* RpoT were appended to the N-terminus of GFP and the construct expressed in *Saccharomyces cerevisiae*, the resulting green fluorescence displayed a reticulate network typical of mitochondrial morphology in live yeast cells (Fig. 3A). To confirm that this fusion protein was targeted to the mitochondrion, an immuno-fluorescence assay was performed on fixed cells using an antibody raised to the beta subunit of ATP synthase as a mitochondrial marker (Fig. 3B). Superposition confirmed that the GFP signal co-occurs with the mitochondrial signal (Figure 3C). This result is consistent with the *H. triquetra RpoT* gene presequence encoding a mitochondrial RNA polymerase.

### Search for other rpoT Sequences

Since 5′-RACE gave only one sequence, we turned to genomic sequencing to see if we could find any evidence for alternative *RpoT*-like genes. Although the C-terminal half of RpoT has the most highly conserved sequence blocks (numbered 5–10 in Fig. 2A), there are regions between blocks that are not conserved at all. It might therefore be possible to find evidence for one or more additional genes by genomic amplification across the non-conserved segments using primers based on the conserved regions. The three primer pairs (f2550/r2750, f2750/r3100, p2f1/p2r1) were designed in such a way that each genomic PCR product would span an unconserved region (Fig. 2A). The first pair spanned a large variable region between blocks 5 and 6A, the second covered most of blocks 6a–8 with smaller variable regions in between, and the third covered blocks 8–10. Even using a variety of annealing temperatures, each of the primer pairs gave only one unique product.

**Figure 4:**
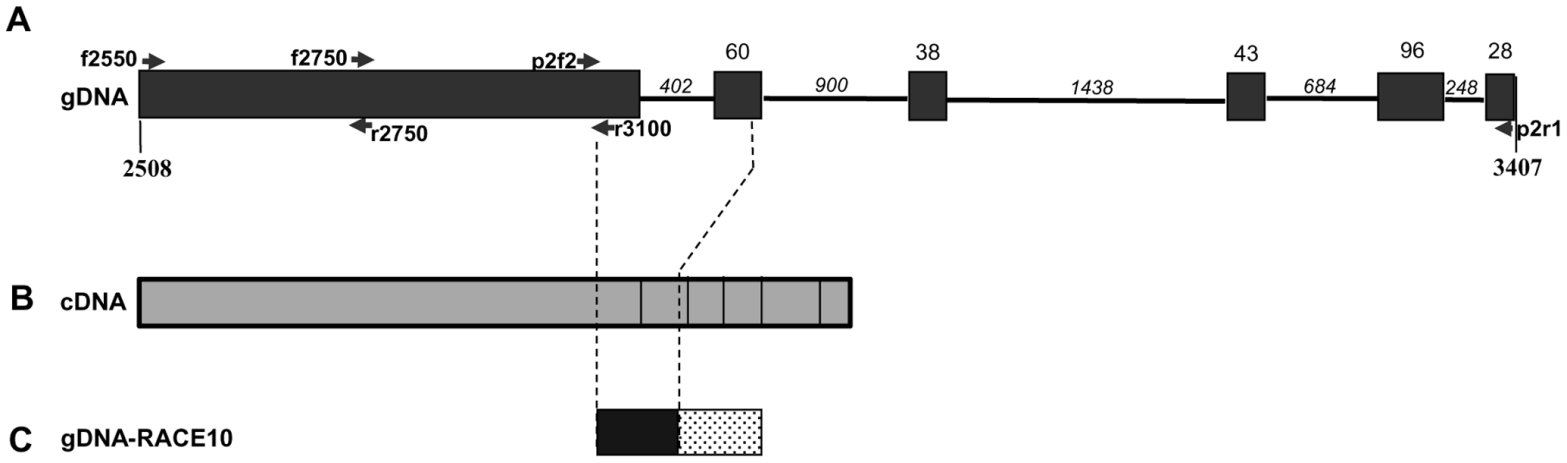
Exon-intron organization of Blocks 5–10 in *H.* *triquetra rpoT*. A. Schematic of genomic DNA sequence determined by RT-PCR showing location of the three primer pairs (f2550/r2750, f2750/r3100 and p2f2/p2r1; Table S2). Boxes, exons; lines, introns. (not to the same scale). B. Schematic of the cDNA sequence. (C) Genomic DNA encoding RACE10 transcript has lost the first intron, suggesting it originated by reverse transcription of a mRNA followed by integration into the genome. Numbers in A (2508, 3407) are nucleotide numbers.

The genomic sequences obtained from the first two primer pairs (encompassing blocks 5,6,7 and part of 8) exactly matched the cDNA sequences, i.e. there were no introns in this region (Fig. 4A), and no evidence for a related but different gene. The third primer pair (p2f2/p2r1), spanning from 3025 to 3407 of the cDNA sequence (blocks 8–10 of the protein sequence), produced a band of about 4 kb, which was cloned. DNA from 20 colonies all showed the same restriction digest pattern with three restriction endonucleases (*Bam*HI, *Kpn*I, *Xba*I). When one clone was sequenced and aligned with the cDNA sequence, it showed the presence of five large introns (402 bp, 900 bp, 1438 bp, 684 bp and 248 bp) separated by small exons of 60 bp, 38 bp, 43 bp and 96 bp (Fig. 4A). Each of the five introns has a canonical 3′ splicing site dinucleotide AG, but at the 5′ end they appear to be less conserved, with GC (3 times), GA (1) and GT (1).This is consistent with what has been found in other dinoflagellate species [1].

### Variant 3′-ends

**Figure 5:**
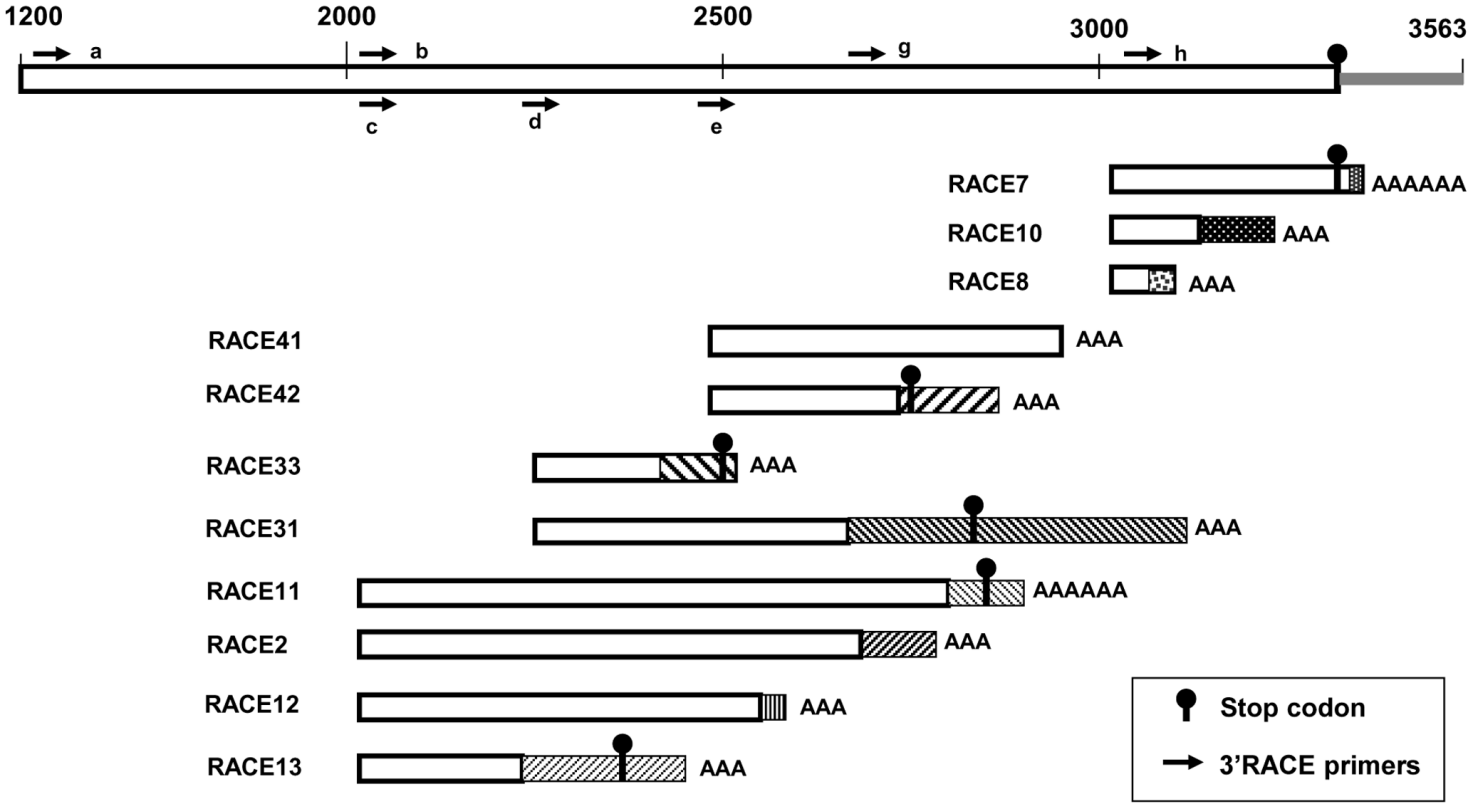
Different 3′ ends of poly(A)-tailed mRNAs. Part of the full-length cDNA sequence is shown on the top line (original 3′-UTR grey); the various poly(A)-tailed transcripts are shown below. White sections are at least 99% identical to the full-length sequence; dotted and hatched sections represent novel sequences. Primer ‘a’ was used as outer forward primer amplifying RACE11, RACE12 and RACE13 with primer ‘b’ as inner primer. Primers ‘b’ and ‘c’ were used to amplify RACE2; primers ‘c’ and ‘d’ for RACE31 and RACE33; primers ‘d’ and ‘e’ for RACE41 and RACE42, and ‘g’ and ‘h’ for RACE7, 8 and 10. Primer sequences are listed in Table S2.

The assembled 3563 bp sequence included a 151 bp 3′-UTR sequence that did not end with a poly(A) tail. Another 3′ RACE with two rounds of nested PCR using outer primer p2f1(g) and inner primer 3′endf1(h) (Fig. 5, Table S2) gave three different products (RACE7, 5 clones; RACE10, 2 clones; RACE8, 1 clone) which were shorter and had small segments (20 bp, 103 bp and 32 bp) of unique sequence at their 3′ ends (Fig. 5). In all the products, the rest of the sequence was at least 99% identical to the corresponding part of the original sequence. We did not find the original 3′-UTR sequence with a poly(A) tail. Among the three novel variants, only RACE7 maintained the original translation reading frame with a stop codon. If translated, the products of the genes encoding RACE10 and RACE8 would be missing most of block 8 and all of blocks 9 and 10 and would not be catalytically active [42]–[44]. To see if there were even shorter poly(A)-tailed transcripts or transcripts differing from the original sequence, 3′ RACE was done using primers farther upstream. Another eight *rpoT* transcript variations were retrieved, only one of which (RACE41) completely matched the original sequence. The other sequences were even more truncated and had novel 3′ end sequences (Fig. 5). Five of them had stop codons (TGA or TAA) at different distances from the poly(A) tail.

In order to determine whether the variant 3′ ends were the result of post-transcriptional modification of different regions of the same transcript or whether these transcripts came from other (partial) gene copies, we did genomic PCR for four transcript variations (RACE10, RACE11, RACE13 and RACE33) by using forward primers located in the regions identical to the original sequence and reverse primers in the novel regions. All the genomic sequences were identical to their mRNA counterparts. This result suggested that these short transcripts were each derived from a unique gene and were not alternative transcripts of the full-length gene. A preliminary estimate with quantitative real-time PCR showed that RACE10, RACE13 and RACE33 were highly expressed compared to the full-length genes (data not shown).

The most interesting case was that of RACE10, where the genomic PCR product matched the cDNA product, i.e. without the intron of the original genomic sequence (Fig. 4 B,C). It suggested that this particular gene might have originated by reverse transcription of an aberrant mRNA followed by insertion into the genome [45].

## Discussion

The sequence of the *H. triquetra RpoT* gene encodes a typical mitochondrial single-subunit RNA polymerase of the phage T7 family [14], [22], [37], with a presequence that is able to target a GPF construct into the mitochondrion of *S. cerevisiae*. RNA polymerases of the phage T7 type fold into two roughly globular domains: a variable N-terminal domain primarily involved in promoter binding and initiation of transcription, and a relatively conserved C-terminal domain that contains the catalytic site [21], [22], [43], [44]. Alignment of the dinoflagellate RpoT protein sequence with other publically available sequences showed that its C-terminal domain has the 9 conserved sequence blocks (numbered 2–10) found in all T7-type polymerases [37], including the invariant amino acids known to be essential for catalytic activity [21], [43]. Overall, 50 residues are conserved among all these sequences if phage T7 is included, and 72 if only complete mitochondrial polymerase sequences are included.

A maximum likelihood phylogenetic tree that included partial RpoT sequences from a second dinoflagellate, *Lingulodinium polyedrum* (Fig. 2B) showed that the dinoflagellate sequences were most closely related to those of apicomplexans such as *Plasmodium*, followed by the basal non-photosynthetic dinoflagellate *Perkinsus* and the basal but photosynthetic apicomplexan *Chromera*, as would be expected given the sister-group relationship of dinoflagellates and apicomplexans. The plastid-targeted RpoT sequences of plants were not included in the tree because the gene duplication that gave rise to them happened after the divergence of the Plantae from the Alveolata [37]. Plants appear to be the only group that has duplicated its mitochondrial gene and retargeted the product to the plastid, and on phylogenetic trees the plastid copies cluster with the plant mitochondrial copies to the exclusion of the single mitochondrial gene in green algae [36].

When the dinoflagellate C-terminal domain (from position 501) was modeled on the x-ray crystal structure of human mitochondrial rpoT using the Phyre 2 program [34], the two structures were almost superimposable, with 41% sequence identity. This predicts that the catalytic mechanism of the dinoflagellate polymerase will be very similar to those of the well-studied bacteriophage, yeast and human polymerases [44]. In contrast, the N-terminal domain of RpoT, involved in promoter binding and initiation of transcription, shows very little cross-species conservation. When we examined our sequence alignments in this region of the protein, we found several small blocks that corresponded to alpha helices D, F, and I of the crystal structure [21], [22]. The human mitochondrial and phage T7 polymerase crystal structures show similarities in folding of this domain even in the absence of sequence relatedness [22], but it would be premature to draw any conclusions in the absence of functional information. The story is further complicated by the fact that all the RpoT’s except those of bacteriophages require one or more additional protein factors for successful initiation of transcription [18], [22], [44], [49]. It is therefore possible that evolutionary changes of the dinoflagellate N-terminal domain would have co-occurred with changes in the number or roles of such factors, which are unlikely to have evolved in parallel in the animal and alveolate lineages.

We did not find any evidence for another intact *RpoT* gene that might be targeted to the plastid in *H. triquetra*. There is accumulating evidence that all dinoflagellate nuclear gene transcripts have spliced leaders [50], so 5′-RACE with the SL primer should have detected a second transcript that included a bipartite targeting motif for plastid import, rather than the unique sequence that was obtained. The RACE7 sequence obtained by 3′-RACE suggests that there are two functional copies of the *RpoT* gene which differ only in the 3′-UTR. The gene with the long 3′-UTR and no poly(A) tail contains a putative polyadenylation signal (AAAAAC) 94 bp downstream of the stop codon, identical to the consensus sequence found in several dinoflagellate species [47]. However the shorter RACE7 transcript, which has a tail, does not have any sequence resembling the putative polyadenylation signal immediately prior to the polyadenylation site. Other dinoflagellate species do not show any common polyadenylation motif [48].

Another possibility is that a single gene could encode two potential initiation codons, as found in the *Physcomitrella patens RpoTmp* gene; one producing a protein targeted to plastids and the other a protein targeted to mitochondria [46]. The *H. triquetra RpoT* gene has several downstream AUG codons, some in-frame and some in the other two translation frames. The deduced protein sequences were examined for possible ER signal sequences, which would be required for the first step of targeting to a secondary plastid [41]. No potential signal sequences were detected. This argues against the possibility that a different translation initiation site or a frameshift might generate a plastid targeted RpoT polypeptide.

We were not able to find any trace of a typical plastid-encoded multisubunit RNA polymerase by degenerate PCR with primers derived from a conserved region of the plastid *rpoB* gene (data not shown). We had predicted that these genes (*rpoB*, *rpoC1* and *rpoC2*) might have been transferred to the nucleus, like *rpoA* and most of the usual plastid genes [1], [2], since none of them have been found on plastid DNA minicircles. However, dinoflagellate gene sequences are often very divergent [2], [13], so it is possible that whichever polymerase is responsible for plastid transcription simply has not been detected. The answer to this problem may be revealed in the course of the ongoing genome sequencing projects.

The variety of truncated poly(A) tailed transcripts found by 3′-RACE appear to be the products of independent (defective) genes. In each case, they are missing enough of the coding sequence so that any protein product would not be catalytically active. Bachvaroff and Place [47] noted that 10 of 46 actin genes in *Amphidinium carterae* were pseudogenes. In the case of RACE10, genomic sequencing suggested that a truncated mRNA had been reverse transcribed and then integrated into the genome, because its genomic sequence did not contain the intron found in the full-length gene. All the truncated genes were at least 99% identical to the full-length gene except for the novel 3′-end sequences, suggesting that they might also have originated by retroposition. It has been suggested that sequential retroposition involving the conserved spliced leader sequence has been an important factor in the origin of multicopy gene families in dinoflagellates [45], [48],

Some highly expressed dinoflagellate genes are found in multi-copy tandem arrays, although whether they all produce polycistronic transcripts is still being debated [1], [30], [47], [48]. We did not find any evidence for tandem copies of the *RpoT* gene, consistent with its being a gene generally expressed at low levels [37], nor did we find evidence for polycistronic transcripts.

Mitochondrial transcription in dinoflagellates might be expected to be unusual. The numerous copies of genes and gene fragments that characterize dinoflagellate mitochondrial genomes present a challenge to generating a functional transcriptome. Furthermore, precise 5′ transcript termini are required for trans-splicing of *cox3*, and precise 3′ positions of polyadenylation are necessary to generate stop codons, indicating little room for error in this process [4], [51]. RNA-seq data indicates that polycistronic transcripts of mitochondrial sequence exist in dinoflagellates, suggesting that either few transcription initiation sites, or limited control of transcription initiation, might be sufficient to generate a pool of mitochondrial RNAs [4], [9]. Northern blot analyses of mitochondrial genes, however, only detect RNAs consistent with coding sequence of individual genes, and do not detect polycistronic species [4], [9]. Therefore it is currently unknown whether polycistronic transcripts are rapidly and accurately processed down to their single gene forms, or if a highly precise transcriptional machinery itself is able to decipher the genetic ‘wheat’ from the ‘chaff’ that apparently dominates these very complex mitochondrial genomes.

## Supporting Information

Table S1 Primer sequences for initial cloning of *rpoT*.
(DOC)

Table S2 Primer sequences for 5′- and 3′-ends of *rpoT* gene.
(DOC)

Table S3 Sequences used in Figure 2B.
(DOC)
