# Supplementary material for: Mitochondrial Genes of Dinoflagellates Are Transcribed by a Nuclear-Encoded Single-Subunit RNA Polymerase
Source: PLoS One. 2013 Jun 19;8(6):e65387. doi: 10.1371/journal.pone.0065387 (PMC3686807; doi:10.1371/journal.pone.0065387)
Supplement: Table S1 — Primer sequences for initial cloning of rpoT. (DOC) [file pone.0065387.s001.doc]

Table S1. Primer sequences for initial cloning of *rpoT*.

| Degenerate RT-PCR for *rpoT* | |
| --- | --- |
| rpoTdf1 | GITSITGCAACGGIYTNCARCA |
| rpoTdf2 | GGTIGTIAAGCAGACNGTNATGAC |
| rpoTdr | GCGTGIGTCCARWAISWRTCRTG |
| Inverse RT-PCR for *rpoT* | |
| rpoTinvr1 | CCAGCTGCCGCGTTTCTTCGA |
| rpoTinvr2 | GGCCTCCCTCTCAACCTTCTC |
| rpoTinvr3 | GTCGCTGGGCGTGAGGTTCA |
| rpoTinvr1 | GGTGGAACTGCCTTGGACG |
| rpoTinvr2 | ATCGCCGTCGCACCTTCT |
| 5' RACE for *rpoT* | |
| rpoTr1 | ACAGGATTGGCCTCCCTCTCAAC |
| rpoTr2 | AGACCATTGCAGGTTCCGTCAAG |
| rpoTr3 | GACTCGCGCATCAAAGGTGATCT |
| rpoTr4 | GAGCACACGGATCTCCCTCTT |
| rpoTr5 | AGCATCTGGTTCAGCACCGTA |
| rpoTr6 | CTTCACTTCGTTGTCCGCATC |
