# Supplementary material for: Mitochondrial Genes of Dinoflagellates Are Transcribed by a Nuclear-Encoded Single-Subunit RNA Polymerase
Source: PLoS One. 2013 Jun 19;8(6):e65387. doi: 10.1371/journal.pone.0065387 (PMC3686807; doi:10.1371/journal.pone.0065387)
Supplement: Table S2 — Primer sequences for 5′- and 3′-ends of rpoT gene. (DOC) [file pone.0065387.s002.doc]

**Table S2. Primer sequences for 5'- and 3'-ends of *rpoT* gene.**

| **Name** | **Sequence** | **Position on cDNA** |
| --- | --- | --- |
| **Uni 5’ Ht** | DCCGTAGCCATTTTGGCTCAAG | 1-22 |
| **r82** | ACTGCTTGCAGAGCCGCTGGAT | 580-601 |
| **r81** | GTGGCAGCCAAGCAGCTCAGT | 669-689 |
| **p2f1 (g)** | GCAGCGCAAGGTCGTCAA | 2708-2722 |
| **3’endf1 (h)** | GCTTCCCGCCCAACTTTATC | 3100-3119 |
| **f1200 (a)** | CCGTCGGGGATGCTGTGGAGAATG | 1237-1260 |
| **p1f1 (b)** | AGCGCATATACTTCCCGCA | 2032-2040 |
| **f2050 (c)** | ACTTCCCGCACAACGTTGACTTCAG | 2041-2065 |
| **f2300 (d)** | TGGCCGAGGAGCACCTCAAGTTCTG | 2290-2314 |
| **f2550 (e)** | AGCGACCGACCGCAGGATGTCTACA | 2508-2532 |
| **r2750** | TGGTGACACCGTAGCAGATCGTCAT | 2757-2733 |
| **f2750** | TCGTCAAGCAGACGGTTATGACGAT | 2716-2740 |
| **r3100** | GAGGCATCGAGCGAGTGGATAAAGT | 3112-3136 |
| **p2f2** | CAAAGCAGCGGATGGGCTT | 3085-3103 |
| **p2r1** | GCCAAAGATGTACTTGCTCTGG | 3386-3407 |
| **3RACE10-f1** | CTCGATGCCTCGCACATGATGATG | 3126-3149 |
| **3RACE10-r1** | GTGCTTTGAACGACACTCCTGCAT | Unique segment |
| **3FRC11-r1** | CGTCTGAATCTCGTTCAGGTGTATA | Unique segment |
| **3FRC13-r1** | CGCGTCAGACTCAGTCGGGATTCCA | Unique segment |
| **3FRC33-r1** | AGCCGTCACCGCGGACCCTACGAT | Unique segment |
