# Supplementary material for: Mitochondrial Genes of Dinoflagellates Are Transcribed by a Nuclear-Encoded Single-Subunit RNA Polymerase
Source: PLoS One. 2013 Jun 19;8(6):e65387. doi: 10.1371/journal.pone.0065387 (PMC3686807; doi:10.1371/journal.pone.0065387)
Supplement: Table S3 — Sequences used in Figure 2B. (DOC) [file pone.0065387.s003.doc]

Table S3. Sequences used in Figure 2B

| Name | Genbank Accession Number | JGI-DOE Protein ID |
| --- | --- | --- |
| *Heterocapsa triquetra* | GU_390406 |  |
| *Aureococcus anophagefferens* |  | Auran1:20280 |
| *Fragilariopsis cylindrus* |  | Fracy1:244620 |
| *Guillardia theta* |  | Guith1:823930 |
| *Phaeodactylum tricornutum* |  | Phatr2:49483 |
| *Phytophthora ramorum* |  | Phyra1:96821 |
| *Pseudonitschia multiseries* |  | Psemu1:318067 |
| *Thalassiosira pseudonana* |  | Thaps3:21462 |
| *Ectocarpus siliculosis* | CBJ30655 |  |
| *Galdieria sulphuraria* | N/A |  |
| *Cyanidioschyzon merolae* | CMJ257C |  |
| *Physarum polycephalum* | ABB71761 |  |
| *Drosophila melanogaster* | NP_608565 |  |
| *Xenopus tropicalis* | XP_002939816 |  |
| *Mus musculus* | NP_766139 |  |
| *Homo sapiens* | NP_005026 |  |
| *Rhizopus delamar* | EIE89866 |  |
| *Phanerochaete carnosa* | EKM52814 |  |
| *Neurospora crassa* | XP_962570 |  |
| *Candida tropicalis* | XP_002550066 |  |
| *Ashbya gossypii* | NP_983626 |  |
| *Saccharomyces cerevisiae* | NP_116617 |  |
| *Micromonas sp (RCC299)* | CAP70041 |  |
| *Ostreococcus lucimarinus* | XP_001419848 |  |
| *Selaginella moellendorffii* | CAP70041 |  |
| *Chenopodium album* | CAA69305 |  |
| *Arabidopsis thaliana* | CAC01769 |  |
| *Zea mays* | AAD22976 |  |
| *Oxytricha trifallax* | AEV66615 |  |
| *Paramecium tetraurelia* | XP_001435950 |  |
| *Tetrahymena thermophila* | XP_001013489 |  |
| *Chromera velia* | JO796029 |  |
| *Perkinsus marinus* | XP_002785982 |  |
| *Lingulodinium polyedrum* | JO764413,JO700446,JO741464 |  |
| *Babesia bovis* | XP_001611431 |  |
| *Plasmodium falciparum* | XP_001347935 |  |
| *P. vivax* | XP_001615369 |  |
| *P. knowlesii* | XP_002259256 |  |
| Bacteriophage T7 | NP_041960 |  |
